# Supplementary material for: Potential Owner-Related Risk Factors That May Contribute to Obesity in Companion Dogs in Aotearoa New Zealand
Source: Animals (Basel). 2022 Jan 21;12(3):267. doi: 10.3390/ani12030267 (PMC8833804; doi:10.3390/ani12030267)
Supplement: Supplementary file 1 [file animals-12-00267-s001.zip › animals-1514169-supplementary.pdf]

Supplementary Table S1. EIT Pet Survey 2019 questions related to demographics, dog diet, and body condition.

Question	Possible Responses
Are you?	Female, Male, Gender diverse
To which ethnic group do you belong? Tick as many as apply:	NZ European, Other European, Māori, Samoan, Cook Island Māori, Tongan, Niuean, Chinese, Indian, don't know, Other (please specify)
To which age range do you belong?	18-24 years, 25-34 years, 35-44 years, 45-54 years, 55-64 years, 65-74 years, 75-84 years, 85 years and over
In which region do you currently live?	Northland, Auckland, Bay of Plenty, Waikato, Taranaki, Gisborne, Hawke's Bay, Manawatu-Wanganui, Wellington, Nelson, Marlborough, Tasman, West Coast, Canterbury, Otago, Southland, Other (please specify)
In the last 12 months, what was your total household income?	Less than $14,000, $14,001–48,000, $48,001–70,000, $70,001–100,000, Over $100,000, Would rather not say
What is your highest completed qualification?	National Certificate level 1, National Certificate level 2, National Certificate level 3, National Certificate level 4, Trade certificate, Diploma or Certificate level 5, Advanced trade certificate, Diploma or Certificate level 6, Teachers Certificate or Diploma, Nursing Diploma, Bachelor Degree, Bachelor (Hons), Postgraduate Certificate/Diploma, Master's Degree, PhD, Other (please specify)
Including yourself, how many adults (over 18 years of age) live at your usual address?	0, 1, 2, 3, Other (please specify)
How many children under 18 years of age live at your usual address?	0, 1, 2, 3, 4, 5, Other (please specify)
Were you born in New Zealand?	Yes, No
Where were you brought up?<br>Tick as many as apply:	In a town or city, On a farm or rurally, On a lifestyle block, Other (please specify)
Where do you live now?	In a town or city, On a farm or rurally, On a lifestyle block, Other (please specify)
Do you own a dog?	Yes, No
How many dogs do you own?	1, 2, 3, 4, Other (please specify)
What do you feed your dog/s?*<br>Tick all that apply:	My dog is fed dog roll, My dog is fed dog biscuits from the supermarket, My dog is fed specialised dog food from a pet shop or vet clinic, My dog is fed raw meat, My dog is fed wet food, My dog is fed table scraps/human food, My dog is fed food that I have cooked for them, My dog is fed treats, Other (please specify)
Health/care section. Please choose the option that most closely describes how you feel about the following statements.<br>Dogs should:<br>• have a specialised diet from a pet shop or vet clinic<br>• have ribs, hips, and a spine that are not visible but are easily felt	Strongly agree, Agree, Neutral, Disagree, Strongly disagree
Please provide further explanation if required.	Strongly agree, Agree, Neutral, Disagree, Strongly disagree

* With regard to the food type/s fed in NZ, the phrase "dog biscuits" refers to dry food or kibble (as opposed to a dry treat). Wet food is typically bought in cans or foil packets and is available as pate or in chunks with jelly, gravy or broth.
